# A new high-throughput method for simultaneous detection of drug resistance associated mutations in *Plasmodium vivax dhfr*, *dhps *and *mdr1 *genes

**DOI:** 10.1186/1475-2875-10-282

**Published:** 2011-09-24

**Authors:** Céline Barnadas, David Kent, Lincoln Timinao, Jonah Iga, Laurie R Gray, Peter Siba, Ivo Mueller, Peter J Thomas, Peter A Zimmerman

**Affiliations:** 1 Vector Borne Diseases Unit, Papua New Guinea Institute of Medical Research (PNGIMR), Goroka, Papua New Guinea; 2 Infection and Immunity Unit, Walter & Eliza Hall Institute of Medical Research, Parkville, Australia; 3 Department of Mathematics, Case Western Reserve University, Cleveland, USA; 4 Center for Global Health and Diseases, Case Western Reserve University, Cleveland, USA; 5 Centre de Recerca en Salut Internacional de Barcelona (CRESIB), Barcelona, Spain

## Abstract

**Background:**

Reports of severe cases and increasing levels of drug resistance highlight the importance of improved *Plasmodium vivax *case management. Whereas monitoring *P. vivax *resistance to anti-malarial drug by *in vivo *and *in vitro *tests remain challenging, molecular markers of resistance represent a valuable tool for high-scale analysis and surveillance studies. A new high-throughput assay for detecting the most relevant markers related to *P. vivax *drug resistance was developed and assessed on Papua New Guinea (PNG) patient isolates.

**Methods:**

*Pvdhfr, pvdhps *and *pvmdr1 *fragments were amplified by multiplex nested PCR. Then, PCR products were processed through an LDR-FMA (ligase detection reaction - fluorescent microsphere assay). 23 SNPs, including *pvdhfr *57-58-61 and 173, *pvdhps *382-383, 553, 647 and *pvmdr1 *976, were simultaneously screened in 366 PNG *P. vivax *samples.

**Results:**

Genotyping was successful in 95.4% of the samples for at least one gene. The coexistence of multiple distinct haplotypes in the parasite population necessitated the introduction of a computer-assisted approach to data analysis. Whereas 73.1% of patients were infected with at least one wild-type genotype at codons 57, 58 and 61 of *pvdhfr*, a triple mutant genotype was detected in 65.6% of the patients, often associated with the 117T mutation. Only one patient carried the 173L mutation. The mutant 647P *pvdhps *genotype allele was approaching genetic fixation (99.3%), whereas 35.1% of patients were infected with parasites carrying the *pvmdr1 *976F mutant allele.

**Conclusions:**

The LDR-FMA described here allows a discriminant genotyping of resistance alleles in the *pvdhfr*, *pvdhps*, and *pvmdr1 *genes and can be used in large-scale surveillance studies.

## Background

*Plasmodium vivax *is the most widespread of the malaria parasites infecting human hosts and may be responsible for up to 400 million infections each year [1,2]. The widespread belief that this parasite causes only benign malaria has been challenged recently by reports of severe pathology, including cerebral malaria, acute respiratory distress, acute renal failure, and severe anaemia in patients with *P. vivax *infections [3-6]. In addition, there are increasing worldwide reports of treatment failure after administration of the standard drug regimens, mainly chloroquine (CQ) [7-15]. New drugs are now available, but they are significantly more costly than CQ plus primaquine, the standard treatment for *vivax *malaria for the last 50 years [16]. In order to change their treatment policies based on local evidence, countries need efficient methods to assess potential for resistance to current standard treatments.

Monitoring *in vivo *drug resistance remains challenging due to the ability of *P. vivax *to relapse from long-lasting liver stages. At the same time, the recurrence of parasitaemia due to *de novo *infection can confound evaluation of treatment efficacy. Genotyping of recurrent infections [17] makes it possible to distinguish infection by parasites with different genotypes (new infections) [18,19] from infection by parasites with identical genotypes (whether due to relapse or to recrudescence from blood-stage parasites that survived drug treatment) [20]. Of course, even molecular diagnosis cannot distinguish persistent infection from *de novo *infection by a genetically identical parasite. *In vitro *assays, which should provide drug susceptibility data free from the effects of confounding factors, such as host immunity, are still difficult to conduct because of the lack of stable, continuous *P. vivax *in vitro culture [5,21]. Molecular markers of resistance, therefore, represent a useful tool to monitor the introduction and spread of anti-malarial resistance. For *P. vivax*, these markers include mutations in genes encoding the dihydrofolate reductase (PvDHFR) and the dihydropteroate synthase (PvDHPS) that are involved in drug resistance to antifolates (pyrimethamine) [22], and sulphonamides (sulphadoxine) [23], respectively. The 976F mutation in the gene encoding the multidrug resistance 1 protein (PvMDR1), which has been associated with 4-aminoquinolines (amodiaquine, CQ) resistance in some [21,24] but not all studies [21,25].

Further investigations are needed to assess the predictive value of molecular markers; their use in monitoring of *P. vivax *drug resistance needs to be addressed, geographically, at both a local and a large-scale level [26]. For these reasons, a new assay was developed to detect single nucleotide polymorphisms (SNPs) potentially associated with *P. vivax *drug resistance, in *pvdhfr*, *pvdhps *and *pvmdr1 *genes (total of 23 allelic variants). This post-PCR multiplex assay uses ligase detection reaction and fluorescent-microsphere technologies. A similar approach was previously developed to screen for *Plasmodium falciparum *drug resistance-associated SNPs [27]. The assay was validated using sequenced isolates and a subset of samples from Papua New Guinea (PNG), where CQ resistant *P. vivax *was first described in 1989 [12]. Resistance to sulphadoxine-pyrimethamine (SP) was observed to be low until 2000 [28], when PNG introduced CQ or amodiaquine plus SP as the national first line treatment for both *P. falciparum *and *P. vivax*. Since then, levels of resistance to this combination have increased [24,29]. Here the development and application of this multiplex assay is described; the frequency of *P. vivax *genetic markers associated with multiple drug resistance in PNG patient isolates is presented.

## Methods

### Study population and blood sample collection

Samples were collected between 2006 and 2007 through active detection of malaria infections in a cohort of children 1-4 yrs of age in Ilaita, East Sepik Province, Papua New Guinea [30]. The study was approved by the PNG Medical Research Advisory Council. *Plasmodium vivax *infected monkey blood (SalI and Thai-3 strains) was kindly provided by W. E. Collins (Centers for Disease Control and Prevention, Atlanta, GA). *Plasmodium falciparum *positive samples were obtained from a culture of the 3D7 strain.

### DNA template preparation

DNA was extracted from cell pellets (250μL) using the QIAamp 96 DNA blood kit (Qiagen, Valencia, CA). Genomic DNA was extracted from *P. vivax*-infected monkey blood or from *P. falciparum *culture using the QIAamp DNA blood minikit (Qiagen, Valencia, CA).

### *Plasmodium *species diagnosis

*Plasmodium *species diagnosis was performed using a post-PCR ligase detection reaction - fluorescent microsphere assay (LDR-FMA) as described previously [30,31]. Positive samples for *P. vivax *infections were identified and then assessed for mutations in *pvdhfr*, *pvdhps *and *pvmdr1 *genes.

### Multiplex PCR amplification of *P. vivax dhfr*, *dhps *and *mdr1 *target sequences

A nested PCR was developed to perform multiplex amplification of *P. vivax dhfr*, *dhps *and *mdr1 *target sequences. All Nest 1 reactions were performed in a buffer containing 3 pmol of each primer, 67 mM Tris-HCl (pH = 8.8), 3.3 mM MgSO_4_, 16.6 mM (NH_4_)_2_SO_4_, 10 mM mercaptoethanol, 100 μM (each) dATP, dGTP, dCTP, dTTP, and 2.5 U of thermostable DNA polymerase. Nest 2 reactions were performed with 3 μL of PCR products from the Nest 1 reaction using the same conditions except for the primers (6 pmol). Primer sequences and size of PCR products are given in Table A1 (Additional file 1). PCR amplification was performed under the following conditions: Nest 1 - 94°C for 2 minutes and 35 cycles at 94°C for 30 seconds, 60°C for 30 seconds, 72°C for 3 minutes, and a final extension at 72°C for 5 minutes; Nest 2 - 94°C for 2 minutes and 45 cycles at 94°C for 30 seconds, 63°C for 30 seconds, 72°C for 90 seconds, and a final extension at 72°C for 4 minutes. The specificity of the amplification was evaluated following electrophoresis on a 2% agarose gel in 1× TBE buffer (8.9 mM Tris, 8.9 mM boric acid, 2.0 mM EDTA).

### Ligase detection reaction - fluorescence microspheres assay (LDR-FMA)

A description of the three-step, post-PCR LDR-FMA, procedure has been provided in previous publications [27,31]. After PCR amplification of *pvdhfr*, *pvdhps *and *pvmdr1 *target sequences, products were subjected to a ligase detection reaction between modified upstream allele-specific and downstream conserved sequence primers. Modifications included the addition of unique TAG sequences at the 5' extremity of the upstream sequence-specific primers and 5' phosphorylation/3' biotinylation of the downstream primers. In a second step, hybridization occurred between TAG and anti-TAG oligonucleotide probes bound to fluorescent microspheres. The products of this reaction were then incubated in a solution containing streptavidin-phycoerythrin (SA-PE) to allow binding to the 3' biotin of the conserved sequence primer.

One μL of multiplex PCR product was subjected to the ligation reaction, performed in a solution (15μL) containing 20 mM Tris-HCl buffer (pH 7.6), 25 mM potassium acetate, 10 mM magnesium acetate, 1 mM NAD^+^, 10 mM dithiothreitol, 0.1% Triton X-100, 10 nM (200 fmol) each LDR probe, 1 μl of each PCR product, and 2 U of *Taq *DNA ligase (New England Biolabs, Beverly, MA). Reaction mixtures were initially heated to 95°C for 1 min, followed by 32 thermal cycles at 95°C for 15 s (denaturation) and 60.0°C for 2 min (annealing/ligation). The multiplex LDR product (5μl) was then added to 60μl of hybridization solution (3 M tetramethylammonium chloride [TMAC], 50 mM Tris-HCl [pH 8.0], 3 mM EDTA [pH 8.0], 0.10% sodium dodecyl sulfate) containing 250 Luminex FlexMAP™ microspheres for each allelic set. Mixtures were heated to 95°C for 90 s and incubated at 37°C for 40 min to allow hybridization between SNP-specific LDR products and microsphere-labelled anti-TAG probes. Following hybridization, 6 μl of streptavidin-R-phycoerythrin (Molecular Probes, Eugene, OR) in TMAC hybridization solution (20 ng/μl) was added to the post-LDR mixture and incubated at 37°C for 40 min in Costar 6511 M polycarbonate 96-well V-bottom plates (Corning Inc., Corning, NY). Detection of SNP-specific products (Classification signal = microspheres fluorescence/Reporter signal = SA-PE) was performed through a BioPlex liquid array reader (Bio-Rad laboratories, Hercules, CA). All fluorescence data were compiled into Microsoft Excel spreadsheets using Bio-Rad (Hercules, CA) software, Bio-Plex Manager 4.1.

Sequences of LDR primers are given in Table A2 (Additional file 2). These primers were designed to allow the detection of mutations at five *pvdhfr *codons (57-58-61, 117, 173), four *pvdhps *codons (382-383, 553, 647) and one *pvmdr1 *codon (976), with a total of 23 SNPs detected. LDR primers were designed according to available sequences published in the literature and sequences obtained from PNG isolates. To improve the specificity of the assay, a mismatch (G→A) was introduced in the sequence of the primers (at nucleotide 178, accession no. X98123.1) hybridizing against codons 57-58-61 of *pvdhfr *gene. This allowed a decrease of the allele-specific background observed.

### Obtaining controls and sequencing

*Pvdhfr, pvdhps *and *pvmdr1 *genes were amplified by nested PCR from a subset of samples (n = 6); individual species-specific gene fragments were sequenced after purification using the QIAquick PCR purification kit (Qiagen, Valencia, CA). Additionally, six isolates (genotypes known) kindly provided by D. Ménard (Pasteur Institute, Madagascar) and C. Sibley (University of Washington, Seattle, WA) were amplified for *pvdhfr *gene; PCR products were cloned to obtain positive controls for a selection of mutant *pvdhfr *genotypes: 57I (ata), 58R (agg, aga, cgt), 117N/117T (aac/acc), 173L (ctt). Three isolates were cloned in order to obtain *pvdhps *wild type, single mutant 383G and double mutant 382C-383G genotype controls, and two isolates to obtain *pvmdr1 *Y976 and 976F. Cloning was performed using a TOPO-TA vector cloning system, and Top10F competent cells (Invitrogen), according to the manufacturer's instructions; DNA was extracted using the QIAprep Spin Miniprep kit (Qiagen, Valencia, CA). Sequencing reactions were performed by single-pass bidirectional plasmid sequencing (Agencourt Biosciences, Beverly, MA).

## Results

*Plasmodium vivax *infections were observed in 366 (52%) of 704 samples evaluated by *Plasmodium *species diagnosis with a post-PCR LDR-FMA [31]; 113 samples (30.9%) were co-infected with *P. falciparum*. A LDR-FMA multiplex marker analysis was developed to detect SNPs in *pvdhfr*, *pvdhps *and *pvmdr1 *genes related to anti-malarial drug resistance and used to genotype these 366 *P. vivax *samples.

### Validation of the nested multiplex PCR approach for a simultaneous amplification of *pvdhfr*, *pvdhps *and *pvmdr1 *genes

**Figure 1:**
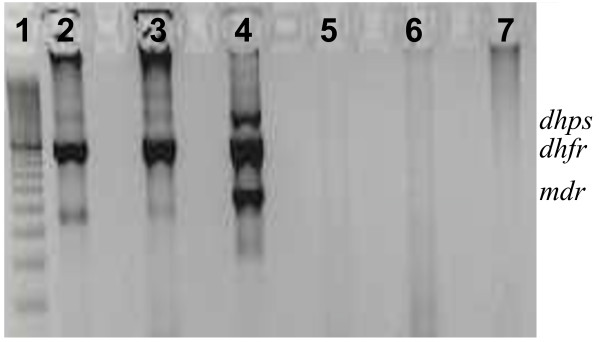
**Gel picture after nested multiplex amplification of *pvdhfr*, *pvdhps *and *pvmdr1 *fragments**. 1: 100 bp DNA ladder. 2: *P. vivax *infected monkey blood (Thai 3). 3 *P. vivax *infected monkey blood (Sal1). 4: *P. vivax *positive PNG isolate. 5: *P. falciparum *3d7. 6: Human DNA (*Plasmodium sp *negative). 7: Blank (water).

To improve the efficiency of the assay, a nested PCR was performed. Three sets of primers were combined in two-step multiplex PCR. Figure 1 shows the fragment sizes obtained (amplification of *P. vivax *Sal-I and Thai-3 strains, and one field isolate), following agarose gel electrophoresis: 1423 bp for *pvdhps*, 917 bp for *pvdhfr *and 545 bp for *pvmdr1*. As mixed *Plasmodium *species infections are commonly observed in PNG, specificity of the amplification was checked using *P. falciparum *3D7 genomic DNA. To evaluate the multiplex PCR amplification strategy, a subset of samples (n = 39) were analysed to determine if all three target sequences were amplified from all samples judged to be positive for *P. vivax *by the species-specific diagnostic assay. From these samples, 37 (95%) produced PCR products of the expected sizes for the three gene-specific target sequences using both single and multiplex PCR strategies.

### Validation of *pvdhfr*, *pvdhps *and *pvmdr1 *LDR-FMA probe specificity

Sequenced isolates or clones were used to check the specificity of LDR product formation and fluorescent signals detected (Table A3, Additional file 3). At the time of this study, a control template for the *pvdhps *553G and 647P mutant alleles was not available.

Eight distinct *pvdhfr *haplotypes were discriminated across codons 57, 58 and 61. Because of the proximity of the three codons, haplotype-specific primers covering the three codons and displaying the different allele combinations were designed. Results show that haplotype-specific background ranged from 168 to 3929 fluorescence units (FU); positive haplotype-specific signals ranged from 6968 to 25348 FU. Independently, three alleles were discriminated at codon 117, with allele-specific background ranging from 374 to 2740 FU, and positive allele-specific signals from 14942 to 24245 FU. Two alleles were discriminated at codon 173, with allele-specific background ranging from 566 to 8083 FU, and positive allele-specific signals from 19770 to 24564 FU.

Four *pvdhps *haplotypes were discriminated at codons 382 and 383 (using haplotype-specific primers). Results show that allele-specific background ranged from 202 to 1798 FU; positive allele-specific signals ranged from 4925 to 21682 FU. Two *pvmdr1 *alleles were discriminated at codon 976. Results show that allele-specific background ranged from 811 to 974 FU; positive allele-specific signals ranged from 15634 to 19634 FU.

**Figure 2:**
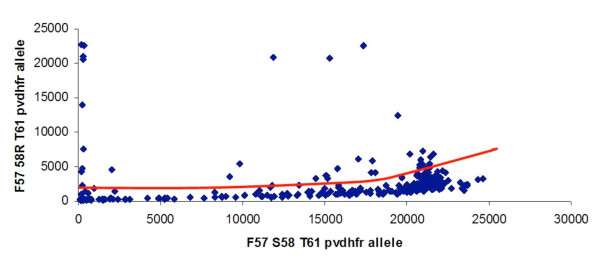
**Increase of the allele-specific background (F57 58R T61 allele signal) observed proportionally to the intensity of another allele-specific signal (F57 S58 T61 allele signal)**.

Similar to what was described elsewhere [32], at some loci, an increase of the allele-specific background was observed proportionally to the intensity of another allele-specific signal (Figure 2). This effect was observed on *pvdhfr *57-58-61 FR(agg)T and *pvdhfr *57-58-61 FR(aga)T allele-specific backgrounds (when *pvdhfr *57-58-61 FST allele-specific signal was high), on *pvdhfr *57-58-61 LR(agg)T allele-specific background (when *pvdhfr *57-58-61 LR(aga)T allele-specific signal was high) and on *pvdhfr *57-58-61 LR(agg)M allele-specific background (when *pvdhfr *57-58-61 LR(aga)M allele-specific signal was high). A similar effect was observed on *pvdhfr *173L allele-specific background (when *pvdhfr *I173 allele-specific signal was high). A more moderate effect was observed on *pvdhps *A647 allele-specific background (when *pvdhps *647P allele-specific signal was high). Interpretation of the fluorescent signals using the polar/multi-dimensional transformation of the data was thus performed.

### Computer-assisted multidimensional data analysis

The coexistence of multiple distinct haplotypes in the parasite population necessitated the introduction of a novel approach to the analysis of LDR-FMA datasets from PNG.

Fluorescence signals from bi-allelic datasets were analysed using a polar transformation of the data in order to improve the data analysis of *Plasmodium *polyclonal infections, as previously described [32]. In the presence of one allele, an increase of the reciprocal allelic SNP signal can be observed, making direct determination of a constant threshold for positivity (presence of the second allele) inappropriate. Briefly, bivariate data were transformed from Cartesian coordinates (x-axis = allele X fluorescence signal; y-axis = allele Y fluorescence signal) into polar (r, θ) coordinates (here $r=\sqrt{x^{2}+y^{2}}$ and θ = arctan(y/x)) to account for the approximately linear crosstalk interaction inducing the background signal. The magnitude of the combined signal, *r*, represents the distance in the plane between a point (x,y) and the origin of the graph. The angle, θ, between the x-axis and a ray extending from the origin to the point (x,y) represents the relative strength of the y *versus *the X signal. Samples with *θ*≈0 correspond to pure infections of type X, samples with *θ*≈90° correspond to pure infections of type Y, and intermediate values of θ representing samples with polyclonal infections. Subsequently, the threshold analysis included two steps. The first step determined a threshold for positivity for infections. The set of samples was divided into bins, from r_min _to r_max_, each having a width of 100 fluorescence units (FU). The threshold of positivity was determined as the value of r corresponding to the bin containing the first minimum (in number of counts) after the first maximum closest to r = 0. In addition, we require that the number of samples in the bin be less than the number that would be expected under the uniform distribution (for a full technical description of the algorithm used for bi-allelic data please see [32]). The second step of the threshold analysis discriminated between polyclonal infections and allele-specific background signal. The set of samples was divided into 45 bins ranging from θ = 0 to θ = π/2 radians (90°). The threshold for the X+/Y- population was set at the first minimum in the histogram as θ increased past the local maximum close to θ = 0 (0°, or horizontal). The threshold for the X-/Y+ population was set at the first minimum in the histogram as θ decreased past the local maximum close to θ = π/2 (90°, or vertical). Upon establishing the thresholds for distinguishing monoclonal versus polyclonal infections in the polar coordinate plane, an inverse polar coordinate transformation returned the data and the diagnosis threshold to the Cartesian plane.

**Figure 3:**
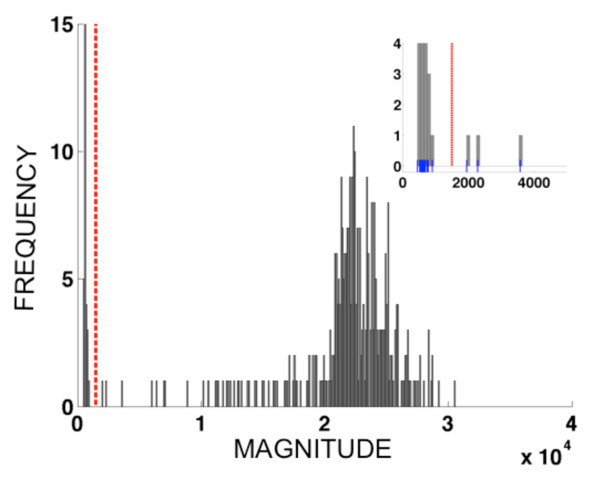
**Histogram showing the distribution of fluorescence vector lengths{*L_i_*:1≤*i*≤*N*} for a multi-allelic data set (*pvdhfr *57-58-61, eight alleles)**. The dashed vertical line indicates the diagnosis threshold for infection *vs*. noninfection. Magnitude threshold = 1500; *N_inf _=*338. Histogram bin width = 100 fluorescence units. Inset panel shows magnified region near the diagnostic threshold, including small vertical blue tick marks indicating the magnitudes for individual samples. Corresponding figures for *pvdhps *382-383 (with four alleles) and for *pvdhfr *117 (with three alleles) are available as Figures S1 and S3, respectively (See Additional Files 4 and 5).

**Figure 4:**
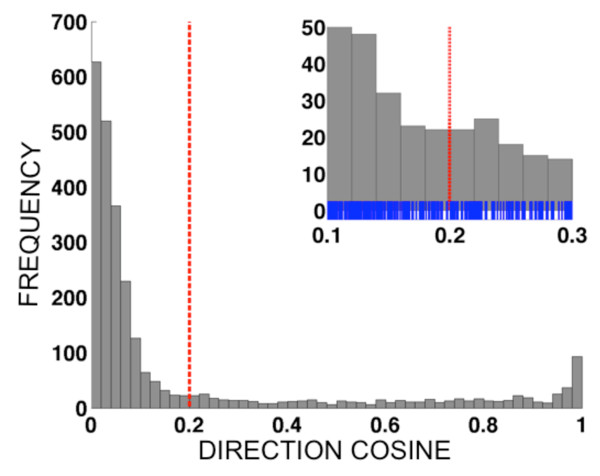
**Histograms showing the distribution of all *N*_inf_×*n *direction cosine values. {*α_ij_*:1≤i≤*N*_inf_,1≤*j*≤*n*} for different multi-allelic data sets**. Only samples judged to be infected based on the magnitude histogram (see Figure 1) are included; the number of infected samples is denoted *N*_inf_, and the number of alleles for a given data set is denoted *n*. The dashed vertical line indicates the diagnosis threshold for infection *vs*. noninfection of individual alleles. For the multi-allellic data sets, the thresholds were determined by visual inspection of the histograms. The *pvdhfr *57-58-61 data contained eight alleles, giving 338 × 8 = 2704 direction cosines total in the histogram; direction cosine threshold = 0.2. Histogram bin width = 0.02. Inset panel shows magnified region near the diagnostic threshold, including small vertical blue tick marks indicating the direction cosines for individual alleles. Corresponding figures for *pvdhps *382-383 (with four alleles) and for *pvdhfr *117 (with three alleles) are available as Figures S2 and S4, respectively (See Additional Files 4 and 5).

Diagnosis of multi-allelic datasets (number of alleles > 2) was accomplished through a computer-assisted procedure analogous to the polar transformation introduced in DaRe *et al *[32]. Each sample was represented as a vector ${\vec{x}}_{i}=\left(x_{i1},\ldots ,x_{ij},\ldots ,x_{in}\right)$ where the first index (*i*), 1≤*i*≤*N*, represented the sample number (out of *N *samples) and the index (*j*), 1≤*j*≤*n *represented the allele (out of *n *alleles). For example, the data set for *pvdhfr *locus 117 comprised *n = 3 *alleles and *N = 366 *samples; the data set for *pvdhps *loci 382-383 comprised *n = 4 *alleles and *N = 366 *samples; the data set for *pvdhfr *loci 57, 58 and 61 comprised *n = 8 *alleles and *N = 366 *samples. The total magnitude of the fluorescence signal for sample *i *was taken to be the Euclidean length of the vector, $L_{i}=\sqrt{\overset{n}{\underset{j=1}{\sum }}x_{ij}^{2}}$. A histogram was constructed of the *N *values of *L_i_*, using bins of width 100 fluorescence units, and the first minimum of the histogram occurring after the first maximum was used as a diagnostic cut-off for distinguishing infected *versus *uninfected samples. Figure 3 shows the histogram of fluorescence vector magnitudes for data from *pvdhfr *57-58-61, along with the threshold obtained. For each sample with vector magnitude exceeding the diagnostic magnitude threshold, the quantities *α_ij_*=*x_ij_*/*L_i _*were calculated. These values, known as *direction cosines*, equal the cosine of the angle *θ_ij _*between the *i*^th ^vector ${\vec{x}}_{i}$ and the *j*^th ^coordinate axis in *n*-dimensional space. When the *i*^th ^sample is infected by parasites carrying at least one allele, but *not *infected by parasites carrying the *j*^th ^allele, the angle *θ_ij _*is close to 90 degrees because the vector lies close to an *(n-1)*-dimensional subspace of the *n*-dimensional space spanned by the *(n-1) *other vector components. Consequently, the vector component *x_ij _*is significantly smaller than those representing the alleles carried by infecting parasites, and the direction cosine *α_ij _*= cos(*θ_ij_*) will be close to zero. In order to assist the practitioner in calling alleles, our data analysis code (written in MATLAB) produces a histogram of all *n*×*N*_inf _values of the direction cosines for a given data set. Figure 4 shows the histogram of direction cosines produced for the *pvdhfr *57-58-61 data set (*n*×*N*_inf _= 8 × 338 = 2704 values).

Once the analysis code produced a histogram, we inspected the histogram visually to identify appropriate diagnostic cut-off values for the direction cosines. Appropriate cut-offs were determined to be 0.20 for *pvdhfr *57-58-61, 0.16 for *pvdhps *382-383, and 0.24 for *pvdhfr *117, see Figure 4, Additional files 4 and 5 (Figure S2 and S4), respectively. Heuristically, the direction cosine threshold was determined by identifying the maximum of the direction cosine histogram closest to *α_ij _*= 0, and *α*_thresh_, the location of the first minimum of the histogram below that maximum, as determined by eye, was used as the cut-off. Allele *j *in sample *i *was scored as *positive *if *α_ij_*>*α*_thresh_, and as *negative *if *α_ij_*≤ *α*_thresh_. Appropriate direction cosine thresholds for these data sets ranged over 0.16≤*α*≤0.24, corresponding to angles of approximately 75°-80°. Please see Figures S1-S4 (Additional files 4 and 5) for additional examples of the magnitude and direction cosine histograms for multi-allelic data sets.

### Application of the LDR-FMA to PNG *P. vivax *samples

All three genes were successfully amplified and genotyped for 305 (83.3%) of the isolates. A positive result for at least one gene associated with drug resistance (*pvdhfr*, *pvdhps *or *pvmdr1*) was obtained for 349 (95.4%) of the isolates in the first run. Of the isolates not successfully genotyped 90% showed lower positivity with the *Plasmodium *species diagnostic assay (500 to 10 000 FU).

Based on *pvdhfr*, *pvdhps *and *pvmdr1 *genotyping, a monoclonal infection was found in only 70/305 (23.0%) samples. The multiplicity of infection (MOI) was determined based on the maximum number of alleles detected at one *pvdhfr, pvdhps *or *pvmdr1 *locus for each isolate and estimated at 2.05 with *pvdhfr *displaying more allelic combinations (MOI = 1.99) than *pvdhps *(MOI = 1.03) and *pvmdr1 *(MOI = 1.29).

Considering only the monoclonal infections (n = 70), 37 isolates (52.9%) displayed a wild-type *pvdhfr *allele (F57-S58-T61-S117-I173). The quadruple mutant genotype (57L-58R-61M-117T-I173) was found in 21 isolates (30.0%); single (n = 2; 2.9%), and double (n = 10; 14.3%) mutant genotypes were also observed (Table 1). All but one of these samples displayed a single mutant type *pvdhps *647P allele, and 12 of them (17.1%) the *pvmdr1 *976F mutation.

**Table 1:** *Pvdhfr, pvdhps, pvmdr1 *genotypes prevalence in *P. vivax *monoclonal infections

	Number of *P. vivax *monoinfected samples (%)
***dhfr *genotype (codons 57/58/61-117-173)**	
**wild type:**	
FST-S-I	37 (52.9)
**double mutant type:**	
F**R**T-**N**-I	2 (2.9)
**LR**T-S-I	10 (14.3)
**quadruple mutant type:**	
**LRM**-**T**-I	21 (30.0)

***dhps *genotype (codons 382/383-553-647)**	
**wild type:**	
SC-A-A	1 (1.4)
**single mutant type:**	
SC-A-**P**	69 (98.6)

***mdr1 *genotype (codon 976)**	
**wild type:**	
Y	58 (82.9)
**mutant type:**	
**F**	12 (17.1)

When considering all infections (Table 2), the prevalence of multiple mutant SNP *pvdhfr *alleles was high. Of 305 infected patients, 223 (73.1%) carried parasites with a wild-type genotype at codons 57, 58 and 61, whereas 200 (65.6%) displayed at least a triple mutant genotype, most likely associated with the 117T mutation, as this mutation was found in the isolates infecting 206 (67.5%) patients. Only one patient carried parasites with the 173L mutation. Interestingly, 303 parasite isolates (99.3%) carried the mutant *pvdhps *647P allele, approaching genetic fixation. In contrast, mutant allelic SNPs in codons 382 or 383 were found in the isolates infecting two to three (0.7 to 1%) patients. The mutant allele associated with *P. vivax *chloroquino-resistance, *pvmdr1 *976F, was observed in 107 (35.1%) patient isolates.

**Table 2:** *Pvdhfr, pvdhps, pvmdr1 *genotypes identified in 305 *P. vivax *infected patients

	Number of infected patients (%)
***dhfr *genotype**	
**57-58-61**	
FST	223 (73.1)
FR**T**	28 (9.2)
**LR**T	116 (38.0)
**LRM**	200 (65.6)
	
**117**	
S	260 (85.2)
**N**	12 (3.9)
**T**	206 (67.5)
	
**173**	
I	304 (99.7)
L	1 (0.3)

***dhps *genotype**	
**382-383**	
SC	305 (100)
S**G**	2 (0.7)
**C**C	2 (0.7)
**CG**	3 (1.0)
	
**553**	
A	305 (100)
	
**647**	
A	5 (1.6)
**P**	303 (99.3)

***mdr1 *genotype**	
**976**	
Y	287 (94.1)
**F**	107 (35.1)

## Discussion

Here is described a new high-throughput method to detect SNPs associated with drug resistance of *P. vivax*, simultaneously in *pvdhfr*, *pvdhps *and *pvmdr1 *genes. To improve the efficiency of this assay, a nested PCR approach was chosen which allowed a successful genotyping in 83.3% of the LDR-FMA *P. vivax *positive samples.

The main challenge faced in developing this multiplexed assay was the interpretation of fluorescence data, due to the simultaneous screening of multiple alleles at one locus and the high level of polyclonality (up to four isolates infecting a single patient). As described previously [32], an increase of background (or off-target) fluorescent signal was consistently observed when PCR amplification produced strong gene-specific fragments. A polar transformation of the fluorescent data measured was performed for bi-allelic datasets; the usefulness of this transformation has been shown before [32]. In contrast, datasets with more than two alleles present required a multidimensional transformation of the data, based on the same principle as the polar transformation. This analysis was applied to three sets of multi-allelic data (n>2): *pvdhfr *codons 57-58-61, *pvdhfr *117, and *pvdhps *382-383.

As in the case of bi-allelic data sets, while the coordinate transformation/histogram segmentation approach better accounted for advancing background due to off-target hybridization, it is not a bulletproof algorithm that guarantees 100% accurate diagnosis. Indeed, given the intrinsic variability of any biological measurement process it is unlikely such a perfect algorithm exists. A significant limitation of the heuristic algorithm described here, at least from a theoretical point of view, is that independent determination of the diagnostic thresholds for each locus can lead to a situation in which a given sample is judged to be positively infected at some but not all loci, which is an inconsistent result. For example, the field data analysed here comprised 366 samples, each of which showed a positive diagnosis for *P. vivax *by LDR-FMA [31]. Of these samples, 17, or 5%, were judged not to have enough net fluorescence signal to be considered positively infected based on the overall magnitude of the fluorescence vector for all seven loci interrogated. Of the 349 samples that showed a positive overall infection based on the magnitude histogram analysis, another 12.6% (44 samples) failed to have a positive single-allele diagnosis for any allele at one of the loci, leading to an incomplete haplotype determination. As a practical matter, in these cases the algorithm described under Methods was supplemented by censoring the samples with an incomplete haplotype determination.

A total of 23 alleles were screened simultaneously in the assay, with the possibility of extending the assay towards screening more alleles as 100 microspheres can be detected in the same multiplexed assay on the BioPlex liquid array reader. This high multiplexing capacity significantly reduces costs associated with diagnosing complex arrays of SNPs associated with anti-malarial drug resistance. While it would cost approximately 3 USD to genotype one sample using a real-time PCR assay screening for only four alleles (TaqMan), the costs of the PCR-LDR-FMA are similar to screen one sample for more than 20 alleles. In addition, as multiple loci of a gene are screened simultaneously, it is possible to identify new genotypes. If a positive signal is observed at one locus of the gene, but a negative signal at another, this would demonstrate the positivity of the PCR, and at the same time, the presence of a new allele not targeted by any of the LDR primers present in the reaction. In this case, it would then be necessary to sequence the PCR product in order to confirm the existence of a new genotype. Modifications to the LDR-FMA are then easily achieved by designing new sequence-specific primers.

Due to the wide use of sulphadoxine-pyrimethamine (SP) to treat falciparum malaria, *P. vivax *has been exposed worldwide to this anti-malarial drug. In Papua New Guinea, SP has been added to 4-aminoquinolines (CQ and amodiaquine) to treat *P. falciparum *and *P. vivax *malaria since 2000. It is therefore not surprising that *pvdhfr *and *pvdhps *mutant alleles [23,33-36] were highly prevalent, with 65.6% of infected patients displaying a triple mutant *dhfr *genotype (57L-58R-61M). Because of a high rate of polyclonal infections (83.3%) and a mean MOI of 1.99 calculated from *pvdhfr *genotyping results, it is difficult to reconstruct haplotypes and give more than an estimation of their prevalence. As shown by the results obtained from monoclonal infections, the 117T mutation was associated with the triple mutant haplotype 57L-58R-61M in 100% of the cases. It is therefore possible to predict an overall rate of infection (among monoclonal and polyclonal infected patients) with a quadruple mutant *dhfr *haplotype to occur in about 66% of the cases.

*In vitro *results (using a yeast model to express PvDHFR) have shown a strong correlation between mutations in *pvdhfr *and the amount of drug needed to inhibit the growth in this system. The 50% inhibitory concentration (IC50) of pyrimethamine is increased by 100-460 fold for a *dhfr *double mutant 58R-117N, and more than 500-fold for a quadruple mutant enzyme 57L-58R-61M-117T in comparison to the IC50 measured for the wild-type enzyme [28,34]. Furthermore, the quadruple mutant haplotype has been associated with *in vivo *treatment failure after treatment with artesunate-SP in a study conducted in Papua, Indonesia [37]. Similarly, the quadruple mutant haplotype has been associated with SP treatment failure when compared to lower mutant parasites (triple mutant to wild type) [38]. The *pvmdr1 *Y976F mutation (when found in combination with *pvdhfr *mutations) was associated with treatment failure in a PNG study conducted in 2004-2005 when *P. vivax *infected patients were treated with AQ+SP [39].

Very few reports have been available on the prevalence of *P. vivax dhfr *and *dhps *mutations in PNG. From 19 isolates collected in 1998 in the Wosera (East Sepik Province) [28], only one (5.3%) displayed a double 57L-58R*pvdhfr *mutation. In the same study, of 25 isolates collected in 2000 in Liksul (Madang Province), nine displayed a *pvdhfr *mutant genotype (six were double mutant 57L-58R). More recently, genotyping results for 94 samples collected in three areas from PNG (Simbu, East Sepik and Madang provinces) between 2004 and 2005 showed higher prevalence of mutant genotypes and the appearance of quadruple mutant parasites (*pvdhfr *57L-58R-61M-117T) in the population [24]. This increase is confirmed here with significantly higher rates of patients infected with mutant genotypes: 57L was found in 78.4% of the participants, 58R in 83.0%, 61M in 65.9%, 117N/T in 70.8%, versus, respectively, 59.6%, 67.0%, 20.2% and 40.4% from the earlier study (p < 0.001). Data from PNG isolates on *pvdhps *have not been generated; only one isolate from PNG was previously sequenced, showing a mutation at codon 647 (647P) [40]. As for *pvmdr1*, the 976F mutation was found at a prevalence of 39.4% [24] and remains at the same level here (35.1%) despite known high level of CQ-SP drug resistance in PNG (51.4% after PCR correction [41]). It has already been discussed that 4-aminoquinoline resistance may result from a multigenic process involving different SNPs and/or gene amplification, variation in the level of expression [24,25]; it is therefore harder to correlate the 976F mutation frequency with an increase of the *in vivo *level of drug resistance.

## Conclusions

While Papua New Guinea and other malaria-endemic countries are changing their national malaria treatment policies, and implementing new treatment strategies, it will be important to assess the impact of such strategies on the evolution of *P. vivax *populations. The new high- throughput method described here represents a valuable tool that can be used for local or large-scale screening of *P. vivax *molecular markers of drug resistance.

## Competing interests

The authors declare that they have no competing interests.

## Authors' contributions

CB designed the assay, was responsible of laboratory work, and contributed to data analysis, and contributed to writing the paper. PJT and DK conceived the multi-allelic data analysis method, DK implemented it, and PJT and DK wrote the methods section on computer-assisted data analysis, and contributed to editing the manuscript. The results of the analysis were independently verified by CB and DK. LT and JI participated in the laboratory work and in editing the manuscript. LG contributed to the design of the assay and editing the manuscript. IM and PS helped with data analysis and writing the paper. PAZ supervised laboratory work and the design of the assay, and contributed to data analysis and writing the paper. All authors read and approved the manuscript.

## Supplementary Material

Additional file 1 **Table A1: Sequences of PCR primers**. Click here for file

Additional file 2 **Table A2: Sequences of LDR primers for *P. vivax dhfr, dhps *and *mdr1 *genes**. Click here for file

Additional file 3 **Table A3: Fluorescence signals obtained for *P. vivax *samples (clones or sequenced isolates) of known *dhfr, dhps *and *mdr1 *genotypes**. Click here for file

Additional file 4 **Figures_S1S2**. **Figure S1**: **Magnitude histogram for *dhps *382-383 **(four alleles); magnitude threshold = 1500; *N_inf _=*337. Histogram bin width = 100 fluorescence units. **Figure S2**: **Direction cosine histogram for *dhps *382-383 **(four alleles, 337 × 4 = 1348 direction cosines total); direction cosine threshold = 0.16. Histogram bin width = 0.02. Figures S1-S2 show the magnitude and direction cosine histograms, respectively, for the multi-allelic data set *dhps *382-383, which exhibited four positive alleles within the population. Inset panels show magnification of the region near the diagnostic threshold, including small vertical blue tick marks indicating the magnitudes of individual samples (S1) or values of direction cosines (S2). Compare Figures 1 and 2 of the main text. Click here for file

Additional file 5 **Figures_S3S4**. **Figure S3**: Magnitude histogram for *dhfr *117 (three alleles); magnitude threshold = 1000; *N_inf _=*339. Histogram bin width = 100 fluorescence units. **Figure S4**: Direction cosine histogram for *dhfr *117 (three alleles, 339 × 3 = 1017 direction cosines total); direction cosine threshold = 0.24. Histogram bin width = 0.02. Figures S3-S4 show the magnitude and direction cosine histograms, respectively, for multi-allelic data set *dhfr *117, which exhibited three positive alleles within the population. Compare Figures 1 and 2 of the main text. Click here for file
