# Supplementary material for: A new high-throughput method for simultaneous detection of drug resistance associated mutations in Plasmodium vivax dhfr, dhps and mdr1 genes
Source: Malar J. 2011 Sep 24;10:282. doi: 10.1186/1475-2875-10-282 (PMC3192712; doi:10.1186/1475-2875-10-282)
Supplement: Additional file 1 — Table A1: Sequences of PCR primers. [file 1475-2875-10-282-S1.DOC]

**Additional file 1- Table A1: Sequences of PCR primers**

| **Gene** | **Primer** | **Sequence 5'-->3'** | **Tm**  **(C)** | **Size fragment (bp)** |
| --- | --- | --- | --- | --- |
| *pvdhfr* | pvdhfr_PF | TGTACCCTTCCATAGGGAGT | 56.0 | 1121 |
|  | pvdhfr_PR | AAAGCTGAAGTACACGAGGTC | 56.2 |
|  |  |  |  |  |
|  | pvdhfr_F | CATCACACCCAGTGGAGCTA | 59.7 | 917 |
|  | pvdhfr_R | ACGCATTGCAGTTCTCCG | 61.0 |
|  |  |  |  |  |
| *pvdhps* | pvdhps-PF | ATGAAAAGAAGGGAAAGCAA | 56.6 | 1945 |
|  | pvdhps-PR | ACAGGGGCAAAGTAAATGAA | 57.2 |
|  |  |  |  |  |
|  | pvdhps-1F | CGCATGATAGCCATTGACAT | 59.5 | 1423 |
|  | pvdhps-3R | GGCTCATTTTGAACCTCCAC | 59.5 |
|  |  |  |  |  |
| *pvmdr1* | pvmdr-3F | ACGACATGATCCAAACGACA | 60.0 | 2784 |
|  | pvmdr-5R | CTTATATACGCCGTCCTGCAC | 59.6 |
|  |  |  |  |  |
|  | pvmdr1-4Fbis | TGCTCTTCCTTGTGAGTACGG | 60.4 | 545 |
|  | pvmdr-4R | CCTTTCGAAGGACAGCTTTG | 60.0 |
