# Supplementary material for: A new high-throughput method for simultaneous detection of drug resistance associated mutations in Plasmodium vivax dhfr, dhps and mdr1 genes
Source: Malar J. 2011 Sep 24;10:282. doi: 10.1186/1475-2875-10-282 (PMC3192712; doi:10.1186/1475-2875-10-282)
Supplement: Additional file 2 — Table A2: Sequences of LDR primers for P. vivax dhfr, dhps and mdr1 genes. [file 1475-2875-10-282-S2.DOC]

**Additional file 2 - Table A2: Sequences of LDR primers for *P. vivax dhfr, dhps* and *mdr1* genes**

| **Gene** | **Primer** | **Sequence 5'-->3'** | **FlexMAP microsphere set ID no.** |
| --- | --- | --- | --- |
| *pvdhfr* | 57-58-61 com | phosphate-GACCTACGTGGATGAGTCAAAGTA-biotin |  |
|  | F57-S58-T61 | caatttcatcattcattcatttcaTATGAAGTACTTCAGCTCGATGAC | 35 |
|  | F57-58R-T61 | cttttcatcttttcatctttcaatGAAGTACTTCAGGTCGATGAC | 37 |
|  | F57-58Raga-T61 | ctacttcatatactttatactacaTATGAAGTACTTCAGATCGATGAC | 63 |
|  | 57L-58R-T61 | ctatctttaaactacaaatctaacTATGAAGTACTTGAGGTCGATGAC | 100 |
|  | 57L-58Raga-T61 | ctaactaacaataatctaactaacTATGAAGTACTTGAGATCGATGAC | 80 |
|  | 57L-58R-61M | ctttcaattacaatactcattacaATATGAAGTACTTGAGGTCGATGAT | 43 |
|  | 57L-58Raga-61M | ctatctatctaactatctatatcaATATGAAGTACTTGAGATCGATGAT | 78 |
|  | 57I-58R-61M | tcataatctcaacaatctttctttATATGAAGTACATAAGGTCGATGAT | 68 |
|  |  |  |  |
|  | 117 com | phosphate-CTGGGAGAGCATCCCCA-biotin |  |
|  | S117 | tacactttctttctttctttctttGGTCATGGGGATAAGCAG | 12 |
|  | 117N | ctactatacatcttactatactttGGTCATGGGGATAAGCAA | 14 |
|  | 117T | ctttaatcctttatcactttatcaGGTCATGGGGAGAAGCAC | 17 |
|  |  |  |  |
|  | 173 com | phosphate-TTGGGGGAGCACAAGTTTATA-biotin |  |
|  | I173 | aatcctttctttaatctcaaatcaCTGAAGTACTACAAATGCTTCATCA | 21 |
|  | 173L | ctacaaacaaacaaacattatcaaCTGAAGTACTACAAATGCTTCATCC | 28 |
|  |  |  |  |
| *pvdhps* | 382-383 com | phosphate-CCCTTATGTGGTCCCCAAT-biotin |  |
|  | S382-C383 | tcatcaatctttcaatttacttacGGGGGAATCGTCCGC | 49 |
|  | S382-383G | caatataccaatatcatcatttacGGGGGAATCGTCCGG | 50 |
|  | 382C-383G | aatcaatcttcattcaaatcatcaGGGGAATCGTGCGG | 16 |
|  | 382C-C383 | aatctaacaaactcatctaaatacGGGGGAATCGTGCGC | 76 |
|  |  |  |  |
|  | 553 com | phosphate-CAAAAAGCACGACCAGTCTATT-biotin |  |
|  | A553 | ctataaacatattacattcacatcGGCCTGGGGTTTGC | 69 |
|  | 553G | aatctacaaatccaataatctcatGGCCTGGGGTTTGG | 60 |
|  |  |  |  |
|  | 647 com | phosphate-CCAAACTGACTAACGGTGACTC-biotin |  |
|  | A647 | tcatcaatcaatctttttcactttGGTAAACTGACGAATGGTGAG | 59 |
|  | 647P | aaacaaacttcacatctcaataatGGTAAACTGACGAATGGTGAC | 48 |
|  |  |  |  |
| *pvmdr1* | 976 com | phosphate-CTTCATTTTTATGAGAGTGTTTGCC-biotin |  |
|  | Y976 | ctttctatctttctactcaataatGCTGTACTGACCGGAACGTA | 94 |
|  | 976F | tacactttaaacttactacactaaCTGTACTGACCGGAACGTT | 95 |
