# Supplementary material for: A new high-throughput method for simultaneous detection of drug resistance associated mutations in Plasmodium vivax dhfr, dhps and mdr1 genes
Source: Malar J. 2011 Sep 24;10:282. doi: 10.1186/1475-2875-10-282 (PMC3192712; doi:10.1186/1475-2875-10-282)
Supplement: Additional file 3 — Table A3: Fluorescence signals obtained for P. vivax samples (clones or sequenced isolates) of known dhfr, dhps and mdr1 genotypes. [file 1475-2875-10-282-S3.DOC]

**Additional file 3 - Table A3: Fluorescence signals obtained for *P. vivax* samples (clones or sequenced isolates) of known *dhfr, dhps* and *mdr1* genotypes**

| **Sample's genotype** | ***dhfr* codons 57-58-61** | | | | | | | | ***dhfr* codon 117** | | | | | | | | | | | ***dhfr* codon 173** | |  | |
| --- | --- | --- | --- | --- | --- | --- | --- | --- | --- | --- | --- | --- | --- | --- | --- | --- | --- | --- | --- | --- | --- | --- | --- |
|  | **FST** | **FRT*** | **FRT^¤^** | **LRT*** | **LRT^¤^** | **LRM*** | **LRM^¤^** | **IRM*** | **S** | | | | **N** | | **T** | | | | | **I** | **L** |  | |
| FST/S/I | **23427** | 3929 | 2392 | 462 | 472 | 205 | 184 | 675 | **24131** | | | | 413 | | 1116 | | | | | **20847** | 7344 |  | |
| FST/N/I | **22642** | 2548 | 1095 | 478 | 582 | 207 | 266 | 219 | 1032 | | | | **18870** | | 2271 | | | | | **20784** | 7197 |  | |
| FRT*/N/I | 370 | **14735** | 576 | 954 | 823 | 258 | 171 | 308 | 1110 | | | | **14942** | | 2138 | | | | | **20947** | 6354 |  | |
| FRT^¤^/N/L | 605 | 1851 | **10262** | 504 | 1880 | 171 | 168 | 305 | 1677 | | | | **17639** | | 2455 | | | | | 566 | **24564** |  | |
| FRT^¤^/N/I | 633 | 1748 | **11242** | 289 | 2045 | 217 | 187 | 218 | 1350 | | | | **20482** | | 2740 | | | | | **19773** | 7362 |  | |
| FRT^#^ | 1044 | 1086 | 1137 | 1128 | 2362 | 210 | 261 | 270 | - | | | | - | | - | | | | | - | - |  | |
| LRT**^¤^**/S/I | 207 | 239 | 432 | 3802 | **25348** | 322 | 1758 | 481 | **23618** | | | | 413 | | 865 | | | | | **20357** | 8083 |  | |
| LRM***^¤^**/S/I | 205 | 197 | 302 | 524 | 1415 | **6968** | **10148** | 781 | **24245** | | | | 374 | | 978 | | | | | **19770** | 7736 |  | |
| IRM*/T/I | 222 | 180 | 209 | 587 | 231 | 1915 | 201 | **18454** | 923 | | | | 1866 | | **20938** | | | | | **21084** | 5542 |  | |
| * Arginine (R) at codon 58 was encoded by agg; | | | | |  |  |  |  |  | | | |  | |  | | | | |  | | |  |
| **^¤^** Arginine (R) at codon 58 was encoded by aga | | | | |  |  |  |  |  | | | |  | |  | | | | |  | | |  |
| ^#^ Arginine (R) at codon 58 was encoded by cgt | | | | |  |  |  |  |  | | | |  | |  | | | | |  | | |  |
|  |  |  |  |  |  |  |  |  |  | | | |  | |  | | | | |  | | |  |
| **Sample’s genotype** | ***dhps* codons 382-383** | | | |  | **Sample's genotype** | ***mdr1* codon 976** | |  | | | |  | |  | | | | |  | | |  |
|  | **SC** | **CC** | **SG** | **CG** |  |  | **Y** | **F** | |  | | | | |  | | |  | |  | | |  |
| 382C-383G | 271 | 240 | 238 | **21682** |  | Y976 | **19634** | 974 | |  | | | | |  | | |  | |  | | |  |
| S382 + 382C -383G | 351 | 203 | **12569** | **8711** |  | 976F | 811 | **15634** | |  |  | | | | | |  | | |  | | |  |
| S382-C383 | **18177** | 1798 | 369 | 379 |  |  |  | | | | |  | |  | |  | | |  |  | | |  |
| S382-C383 | **4925** | 333 | 204 | 202 |  |  |  | | | | |  | |  | |  | | |  |  | | |  |
