# Supplementary material for: A new high-throughput method for simultaneous detection of drug resistance associated mutations in Plasmodium vivax dhfr, dhps and mdr1 genes
Source: Malar J. 2011 Sep 24;10:282. doi: 10.1186/1475-2875-10-282 (PMC3192712; doi:10.1186/1475-2875-10-282)

**Additional file 4.**

Figures S1-S2 show the magnitude and direction cosine histograms, respectively, for the multiallelic data set *dhps* 382-383, which exhibited four positive alleles within the population. Inset panels show magnification of the region near the diagnostic threshold, including small vertical blue tick marks indicating the magnitudes of individual samples (S1) or values of direction cosines (S2). Compare Figures 1 and 2 of the main text.

**Figure S1**: Magnitude histogram for *dhps* 382-383 (four alleles); magnitude threshold = 1500; *N_inf_ =*337. Histogram bin width = 100 fluorescence units.


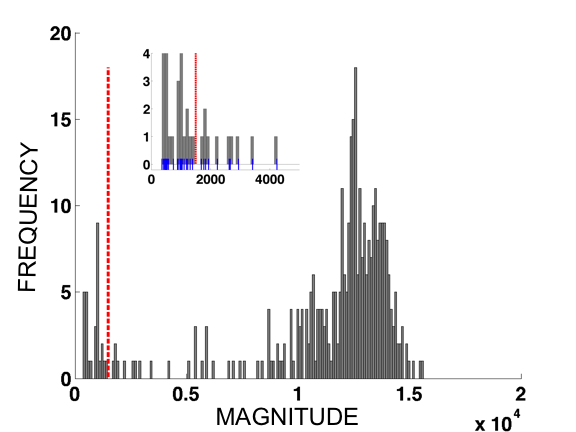


**Figure S2**: Direction cosine histogram for *dhps* 382-383 (four alleles, 337 x 4 = 1348 direction cosines total); direction cosine threshold = 0.16. Histogram bin width = 0.02.


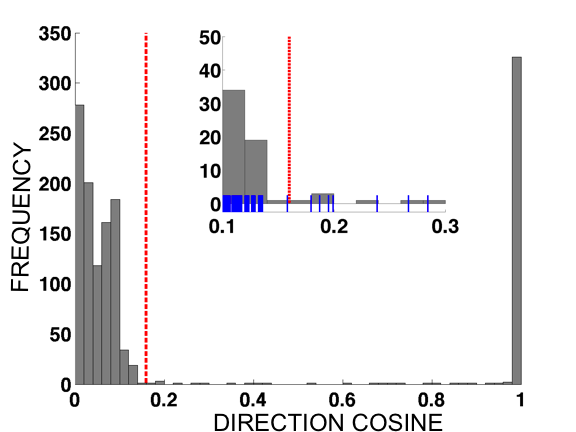

Supplement: Additional file 4 — Figures_S1S2. Figure S1: Magnitude histogram for dhps 382-383 (four alleles); magnitude threshold = 1500; Ninf =337. Histogram bin width = 100 fluorescence units. Figure S2: Direction cosine histogram for dhps 382-383 (four alleles, 337 × 4 = 1348 direction cosines total); direction cosine threshold = 0.16. Histogram bin width = 0.02. Figures S1-S2 show the magnitude and direction cosine histograms, respectively, for the multi-allelic data set dhps 382-383, which exhibited four positive alleles within the population. Inset panels show magnification of the region near the diagnostic threshold, including small vertical blue tick marks indicating the magnitudes of individual samples (S1) or values of direction cosines (S2). Compare Figures 1 and 2 of the main text. [file 1475-2875-10-282-S4.DOC]
