# Supplementary material for: A new high-throughput method for simultaneous detection of drug resistance associated mutations in Plasmodium vivax dhfr, dhps and mdr1 genes
Source: Malar J. 2011 Sep 24;10:282. doi: 10.1186/1475-2875-10-282 (PMC3192712; doi:10.1186/1475-2875-10-282)

**Additional file 5.**

Figures S3-S4 show the magnitude and direction cosine histograms, respectively, for multiallelic data set *dhfr* 117, which exhibited three positive alleles within the population. Compare Figures 1 and 2 of the main text.

**Figure S3**: Magnitude histogram for *dhfr* 117 (three alleles); magnitude threshold = 1000; *N_inf_ =*339. Histogram bin width = 100 fluorescence units.


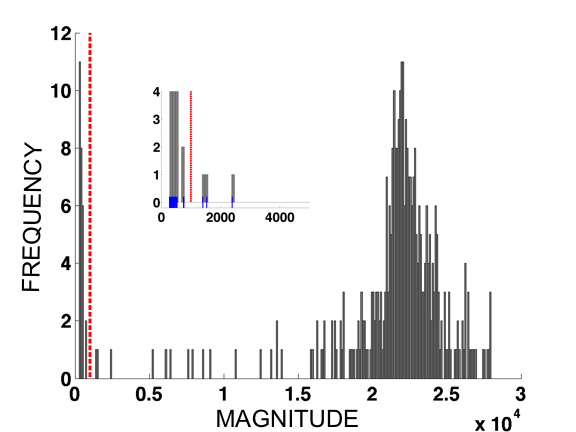


**Figure S4**: Direction cosine histogram for *dhfr* 117 (three alleles, 339 x 3 = 1017 direction cosines total); direction cosine threshold = 0.24. Histogram bin width = 0.02.


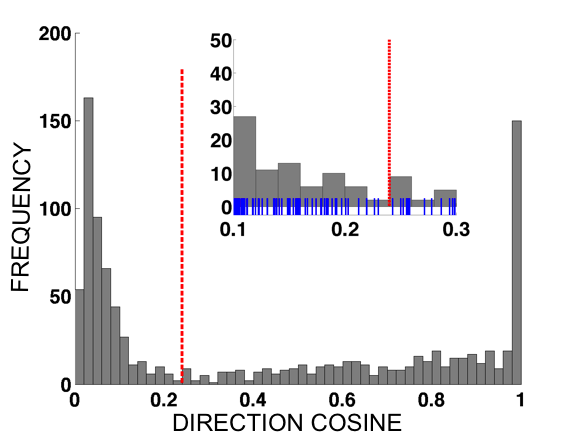

Supplement: Additional file 5 — Figures_S3S4. Figure S3: Magnitude histogram for dhfr 117 (three alleles); magnitude threshold = 1000; Ninf =339. Histogram bin width = 100 fluorescence units. Figure S4: Direction cosine histogram for dhfr 117 (three alleles, 339 × 3 = 1017 direction cosines total); direction cosine threshold = 0.24. Histogram bin width = 0.02. Figures S3-S4 show the magnitude and direction cosine histograms, respectively, for multi-allelic data set dhfr 117, which exhibited three positive alleles within the population. Compare Figures 1 and 2 of the main text. [file 1475-2875-10-282-S5.DOC]
